# Evaluation of an Engineered Zika Virus-Like Particle Vaccine Candidate in a Mosquito-Mouse Transmission Model

**DOI:** 10.1128/msphere.00564-22

**Published:** 2023-02-22

**Authors:** Maria Vittoria Mancini, Rapeepat Tandavanitj, Thomas H. Ant, Shivan M. Murdochy, Daniel D. Gingell, Chayanee Setthapramote, Piyatida Natsrita, Alain Kohl, Steven P. Sinkins, Arvind H. Patel, Giuditta De Lorenzo

**Affiliations:** a MRC–University of Glasgow Centre for Virus Research, Glasgow, Scotland, United Kingdom; b Biologicals Research Group, Research and Development Institute, Government Pharmaceutical Organization, Bangkok, Thailand; c Department of Clinical Pathology, Faculty of Medicine Vajira Hospital, Navamindradhiraj University, Bangkok, Thailand; d Department of Microbiology, Faculty of Medicine, Khon Kaen University, Khon Kaen, Thailand; University of Michigan

**Keywords:** *Aedes aegypti*, arbovirus, flavivirus, mosquito, vaccine, Zika virus

## Abstract

The primary route of Zika virus (ZIKV) transmission is through the bite of an infected *Aedes* mosquito, when it probes the skin of a vertebrate host during a blood meal. Viral particles are injected into the bite site together with mosquito saliva and a complex mixture of other components. Some of them are known to play a key role in the augmentation of the arbovirus infection in the host, with increased viremia and/or morbidity. This vector-derived contribution to the infection is not usually considered when vaccine candidates are tested in preclinical animal models. In this study, we performed a preclinical validation of a promising ZIKV vaccine candidate in a mosquito-mouse transmission model using both Asian and African ZIKV lineages. Mice were immunized with engineered ZIKV virus-like particles and subsequently infected through the bite of ZIKV-infected Aedes aegypti mosquitoes. Despite a mild increase in viremia in mosquito-infected mice compared to those infected through traditional needle injection, the vaccine protected the animals from developing the disease and strongly reduced viremia. In addition, during peak viremia, naive mosquitoes were allowed to feed on infected vaccinated and nonvaccinated mice. Our analysis of viral titers in mosquitos showed that the vaccine was able to inhibit virus transmission from the host to the vector.

**IMPORTANCE** Zika is a mosquito-borne viral disease, causing acute debilitating symptoms and complications in infected individuals and irreversible neuronal abnormalities in newborn children. The primary vectors of ZIKV are Aedes aegypti mosquitoes. Despite representing a significant public health burden with a widespread transmission in many regions of the world, Zika remains a neglected disease with no effective antiviral therapies or approved vaccines. It is known that components of the mosquito bite lead to an enhancement of viral infection and spread, but this aspect is often overlooked when vaccine candidates undergo preclinical validation. In this study, we included mosquitoes as viral vectors, demonstrating the ability of a promising vaccine candidate to protect animals against ZIKV infections after the bite of an infected mosquito and to also prevent its further transmission. These findings represent an additional crucial step for the development of an effective prevention tool for clinical use.

## INTRODUCTION

Zika virus (ZIKV) is an arbovirus belonging to the family *Flaviviridae*. Discovered in Africa in 1947, the virus was then introduced into Yap Island (2007), the South Pacific (2013), and Brazil (later in 2013). Historically associated with relatively mild disease, the most recent epidemics linked ZIKV with severe complications, such as neurological disorders (Guillain-Barré syndrome, encephalitis, and myelitis) (1, 2) and congenital ZIKV syndrome (CZS) (3, 4). ZIKV is mainly a vector-borne virus, but there is strong evidence for sexual (5) and vertical (6) transmission. The dramatic spread of the virus in South America during 2016, and the consequent increase in CZS, led the World Health Organization to declare ZIKV a public health emergency of international concern. Phylogenetically ZIKV strains are divided in two clades, the ancestral African lineage and the subsequent Asian lineage, which was responsible for the 2007, 2013, and 2015 epidemics. It is still an unresolved question how the genetic differences between the two lineages contribute to the associated epidemiology and virulence. Asian lineages are reported to have a high ability to cause persistent infections (7–9); at the same time, it is suggested there is an underappreciation of the real consequences of ZIKV infection in the African continent (10). Currently, there is no effective therapy or vaccine available to control ZIKV infection.

During the past few years, several vaccine candidates have been developed and tested in preclinical stages (11–14), and the most promising ones have now moved to clinical trials (15, 16). Preclinical tests for ZIKV vaccine candidates are performed on murine or nonhuman-primate (NHP) models. The standard procedure involves animal immunization followed by a viral challenge through needle inoculation of laboratory-cultivated ZIKV, normally at a dose range of 10^2^ to 10^4^ PFU (17). The most common routes of infection utilized are intraperitoneal and subcutaneous injection; the latter is thought to mimic vector transmission. While such artificial *in vivo* viral challenges using needle injections ensure accurate dose delivery through defined routes, they do not fully replicate the dynamics of vector-mediated transmission of arboviruses, such as ZIKV, into the human or animal host.

Human transmission of ZIKV occurs mainly when an infected *Aedes* female mosquito (primarily Aedes aegypti and Aedes albopictus) feeds on blood to obtain essential nutrients for egg development. During a blood meal, a mosquito injects saliva containing ZIKV particles, which can infect cells at the bite site and disseminate throughout the vertebrate host. Through the same process, a naive mosquito can also acquire virus present in the bloodstream by feeding on an infected host. To achieve transmission by a mosquito vector, virus present in a blood meal must first cross the midgut epithelium and establish an infection in the salivary glands (SG).

Laboratory-cultured virus stocks may include cell components not commonly present in natural infections, and they therefore represent a possible source of artifacts that may impact the study outcome. In addition, arboviruses naturally replicate in mosquito cells, potentially incorporating vector-specific modifications into virions (e.g., differentially glycosylated surface proteins [18]). Indeed, it has been demonstrated that serial passages in mosquito or mammalian cell lines induce different genetic and phenotypic effects on viral populations (19). It should also be noted that a needle does not inject within the same cellular layers as a mosquito while feeding and probing; moreover, mosquitoes inoculate high doses of virus extravascularly and low doses intravascularly in a live host (20). Most importantly, viral inoculation occurs when mosquitoes deposit saliva as they probe the skin for a blood meal.

Mosquito saliva is an extremely rich molecular cocktail containing a variety of biologically active effectors that facilitate blood feeding by promoting vasodilatation and through modulation of host hemostasis and immune response. Infection of animals with an arbovirus inoculum combined with the vector salivary gland extract (SGE) significantly enhances viremia and pathogenesis (21–23). There is also ample evidence that transmission via mosquito bite is even more effective in enhancing viral infectivity and pathogenicity than needle-mediated inoculations by a mechanism generically (14) defined as mosquito bite enhancement of infection (24, 25). How saliva creates a favorable environment for the first stages of arbovirus infection remains largely unknown, as only a small number of vector saliva proteins have been extensively characterized in the recipient host. It is plausible that the mosquito components affect primary target cells and virus dissemination pathways and modulate host immune responses, creating a favorable environment suitable for arbovirus replication (24, 26–34). In addition to the impact on virulence and viral infectivity, mosquito-mediated infection also alters viral tissue tropism in the host: ZIKV-infected nonhuman primates displayed systemic infections when challenged through mosquito bites, with viral dissemination in the hemolymphatic tissues, female reproductive tract, liver, and kidneys, whereas after subcutaneous needle injection, the virus was detected in the cerebrum and the eyes of some individuals (35).

Viral infection routes are relevant not only for investigating virus dissemination dynamics and tropism in hosts but also for assessing the efficacy of experimental vaccines and therapies against vector-borne pathogens. Virus inoculation through natural vectors includes exposure to factors able to modulate vaccine-induced protective immunity. A promising vaccine candidate against *Leishmania* parasites failed to protect against infected-sandfly challenge, in contrast to the initial promising outcome observed following a needle challenge (36).

We previously reported a promising vaccine candidate comprising ZIKV virus-like particles (VLPs) that have been engineered to display the viral envelope (E) protein locked into a stable dimeric conformation via a disulfide bridge (VLP-cvD) (11). This vaccine conferred protection against both Asian (PRVABC59) and African (MP1751) lineages of ZIKV in a mouse model through the generation of strongly neutralizing antibodies and dramatically reduced virus dissemination to organs, such as brain and testis. Animal challenges, however, were performed with laboratory-cultured ZIKV inoculated through a subcutaneous injection. Here, we further validated this vaccine candidate while taking into account the three key elements of vector-borne pathogen transmission: the virus, the mammalian host, and the mosquito vector. Applying a mosquito-host model, we confirmed the efficacy of the vaccine to protect against vector-transmitted ZIKV disease, and, by using the same transmission system model, we demonstrated its capacity to also impair virus transmission.

## RESULTS

### Protection against an Asian ZIKV strain following vector-mediated infection.

**FIG 1:**
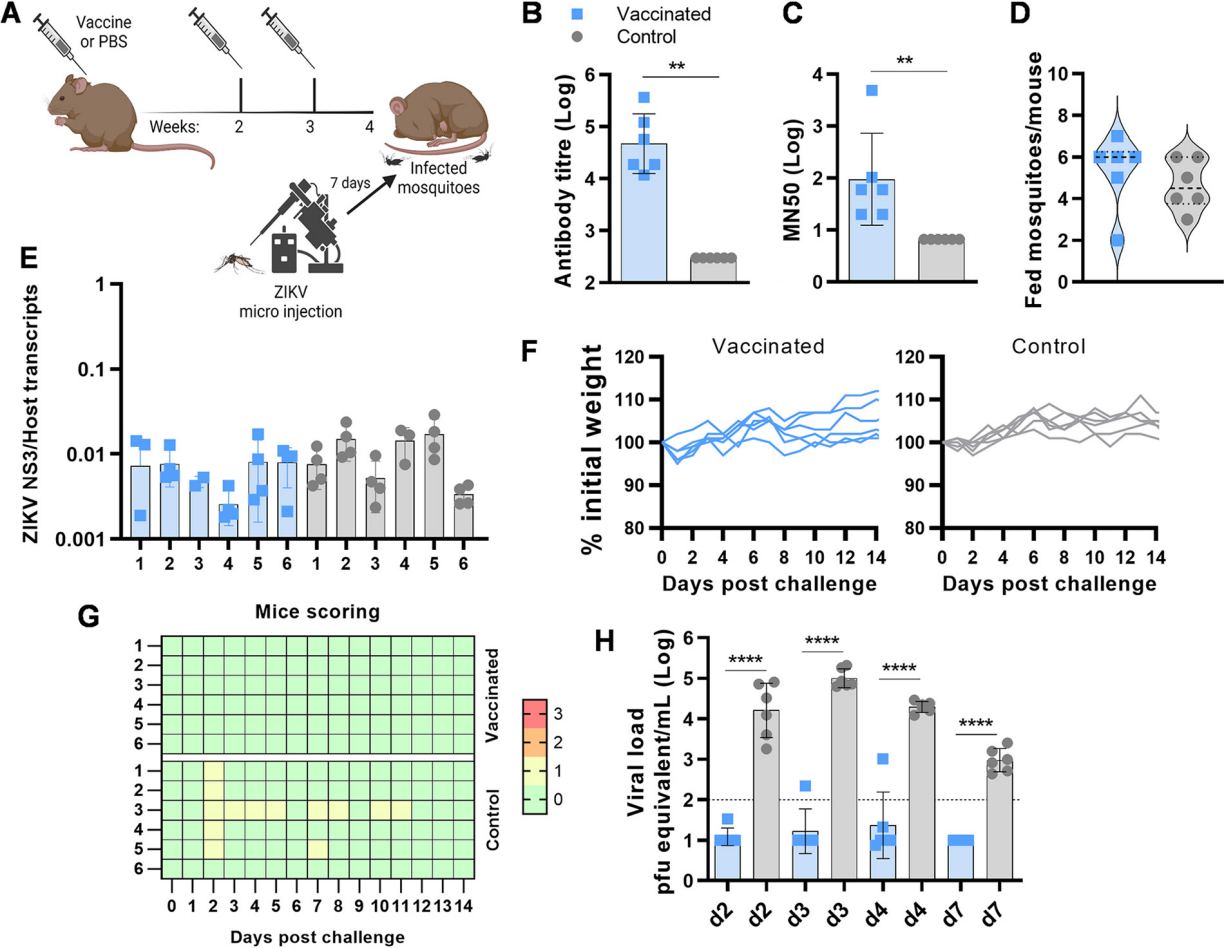
Evaluation of VLP-cvD vaccine efficacy against ZIKV PRVAB59 in a mosquito-mouse transmission model. (A) Schematic representation of the immunization and challenge procedure. Each (*n* = 6) 4-week-old A129 mouse received 3 doses of VLP-cvD or PBS mixed with AddaVax adjuvant. Seven days prior to bite-mediated challenge, mosquitoes were infected with ZIKV by intrathorax microinjection. (B) Anti-dimeric E antibody titers of sera collected from animals immunized with VLP-cvD (blue) or PBS (gray). Antibody titers were determined using ELISA plates coated with mono-biotinylated dimeric E. The titer was defined as the maximum dilution that gives a value higher than three times the value given by the preimmune sera. Control sera were negative at the lowest dilution (1:900), and their titer was calculated as one-third of that dilution (300). Data are geometric means with geometric standard deviations (SD) from three independent experiments. (C) Neutralization of PRVABC59 ZIKV infection. Serially diluted samples of mouse sera were incubated with ZIKV for 1 h before infecting Vero-furin cells. At 72 h postinfection, the intracellular levels of E were determined by capture sandwich ELISA, and the percentage infectivity relative to that of the virus alone was calculated. The results were plotted as MN_50_ values. Data are geometric means with geometric SD from three independent experiments. (D) Number of fed mosquitoes per mouse at the end of the feeding procedure. Mice were anesthetized and put on cardboard cups containing 10 infected mosquitoes each. After 20 min, mice were removed, mosquitoes were anesthetized by exposure to low temperature, and the engorged mosquitoes were counted. (E) Relative quantification of ZIKV genome copies normalized on the host genome in mosquito carcasses. (F and G) Animals were weighed (F) and scored for clinical signs daily postchallenge (G). The scoring system used to monitor animal health following ZIKV challenge was as follows: 0 (green) for no signs of distress or disease, 1 (yellow) for one sign of distress, 2 (orange) for two signs of distress or mild disease, and 3 (red; humane endpoint) for more than two signs of severe disease or loss of 15% of body weight. (H) Viral titers in challenged animals. The levels of ZIKV in the serum at days 2, 3, 4, and 7 postinfection were quantified by RT-qPCR, and the results were plotted as equivalent PFU per milliliter. The limit of quantification was estimated to be 100 PFU/mL, indicated by the dotted line. Data are geometric means from all mice with geometric SD. Assays were performed in triplicate. **, *P* = 0.0021; ****, *P* < 0.0001.

We first aimed to evaluate the efficacy of our ZIKV vaccine against the Asian lineage of the virus in a mosquito transmission mouse model. Two groups each of six A129 knockout (KO) mice (*IFNα*/*βR*^−/−^) received three doses of 2 μg AddaVax-adjuvanted VLP-cvD (vaccinated group) or AddaVax in phosphate-buffered saline (PBS; control group) by subcutaneous injection (Fig. 1A). Separately, 1 week prior to the end of the immunization schedule, 2- to 4-day-old female mosquitoes were intrathoracically injected with a ZIKV strain belonging to the Asian lineage (PRVABC59). Intrathoracic injection was preferred to a standard oral feeding with infected blood to ensure that most mosquitoes acquired the virus.

Analyses of prechallenge sera collected (at week 4 postimmunization) from mice confirmed that, unlike the control group, the vaccinated group had high titers of both anti-ZIKV E and PRVABC59 virus-neutralizing antibodies (Fig. 1B and C, respectively) (*P* = 0.002, Mann-Whitney). We then performed the virus challenge by allowing the PRVABC59-infected mosquitoes to feed on groups of anesthetized vaccinated and control mice. After feeding, fully engorged mosquitoes were counted to estimate the number of individuals that had a complete meal on each mouse (Fig. 1D). An average 40 to 60% feeding rate was achieved. Total RNA was extracted from the carcasses of the engorged mosquitoes and analyzed by reverse transcription-quantitative PCR (RT-qPCR) to confirm the presence of ZIKV (Fig. 1E). It is not possible to determine the precise dose of virus delivered into the host through mosquito bites, nor is it possible to compare and/or correlate viral titer in whole mosquito bodies and the effects of the infection on mice. However, the above-described quantitative analysis of ZIKV titers in each engorged mosquito after the blood meal provides an indication of the overall mosquito viral load and showed no difference between the groups (*P* = 0.08, Kruskal-Wallis test).

After the challenge, mice were monitored daily for 14 days for body weight and clinical signs of infection. Surprisingly, we found the overall effect of infection to be generally mild. Excluding the small loss of weight at 1 day postinfection (dpi), which is most likely due to anesthesia, none of the animals achieved the 10 to 15% weight loss typically associated with ZIKV infection (Fig. 1F). Likewise, no other clinical signs of infection were observed during the entire monitoring period, with all the vaccinated mice displaying no signs of disease (score 0) and the control mice occasionally showing mild signs of distress (score 1) (Fig. 1G). At 2, 3, 4, and 7 dpi, test bleeds were taken, and serum was used to quantify the level of ZIKV in the blood by RT-qPCR. Despite the absence of obvious clinical signs, viremia equivalent to up to 10^5^ PFU equivalents/mL was observed, indicating an active and consistent infection in all control mice (Fig. 1H). In contrast, the viral RNA levels in sera from 3 of 6 vaccinated mice were below the limit of detection (10^2^ PFU equivalents/mL), whereas a small amount of virus with a peak of only 10^3^ PFU equivalents/mL was present in the serum of the remaining one animal.

Altogether, these findings confirmed the capacity of the VLP-cvD to reduce viremia in mice challenged with mosquito-mediated ZIKV PRVABC59 transmission (*P* < 0.0001, two-way analysis of variance [ANOVA]). Since no severe signs of infection were developed by mice, the assessment of the vaccine-conferred protection against lethal infections was not possible to achieve with this specific ZIKV strain.

The lack of visible signs of infection in A129 mice with ZIKV PRVABC59 was also observed during the initial preliminary infection performed to set up the mosquito-mouse infection model (see Fig. S1 in the supplemental material). We reasoned that because AG129 KO mice (*IFNα*/*β*/*γR*^−/−^) lack interferon I and II receptors, they may be more susceptible to ZIKV infection and pathogenesis. Using groups of six animals each, we compared needle injection and mosquito bite as routes of infection, monitoring them for 14 days as described above. An average of 6 engorged mosquitoes per mouse was measured in the mosquito-challenged group (Fig. S2A), which resulted in all being positive for ZIKV infection (Fig. S2B). In both cases, ZIKV PRVABC59 was lethal (Fig. S2C), with no apparent differences in clinical scores (Fig. S2D) and weight loss (Fig. S2E) between the groups. There was no significant difference in viremia between the two groups at 3 dpi (Fig. S2F). These data confirmed that a vector-mediated PRVABC59 infection supports a lethal infection in *IFNα*/*β*/*γR*^−/−^ mice (AG129) but not in *IFNα*/*βR*^−/−^ mice (A129).

10.1128/msphere.00564-22.1 FIG S1 Infection of A129 mice by mosquito feeding. (A) Number of fed mosquitoes per mouse at the end of the feeding procedure. Gray indicates PRVABC59 infected mosquitoes; green indicates naive mosquitoes. (B) Animal body weight variations, calculated as percentage of the initial weight. (C) Clinical scoring for signs of infection. (D) Viral titer in challenged animals at days 2, 3, and 4 postinfection. Data are geometric means from all mice with geometric SD. The dotted line indicates the limit of detection. Download FIG S1, TIF file, 0.3 MB. Copyright © 2023 Mancini et al. 2023 Mancini et al. https://creativecommons.org/licenses/by/4.0/ This content is distributed under the terms of the Creative Commons Attribution 4.0 International license.

10.1128/msphere.00564-22.2 FIG S2 Infection of AG129 mice by mosquito feeding and needle inoculation. (A) Number of fed mosquitoes per mouse at the end of the feeding procedure. (B) Relative quantification of ZIKV genome copies normalized on the host genome from whole mosquito bodies. (C) Survival of mice during the 14-day challenge. Gray indicates mosquito bite-mediated infection; brown indicates needle-mediated infection. (D) Clinical scoring for signs of infection. (E) Mouse body weight variations, calculated as percentages of the initial weight. (F) Viral titer in challenged animals at day 3 postchallenge. Data are geometric means from all mice with geometric SD. The dotted line indicates the limit of detection. Download FIG S2, TIF file, 0.5 MB. Copyright © 2023 Mancini et al. 2023 Mancini et al. https://creativecommons.org/licenses/by/4.0/ This content is distributed under the terms of the Creative Commons Attribution 4.0 International license.

### Protection against African ZIKV strain vector-mediated infection.

**FIG 2:**
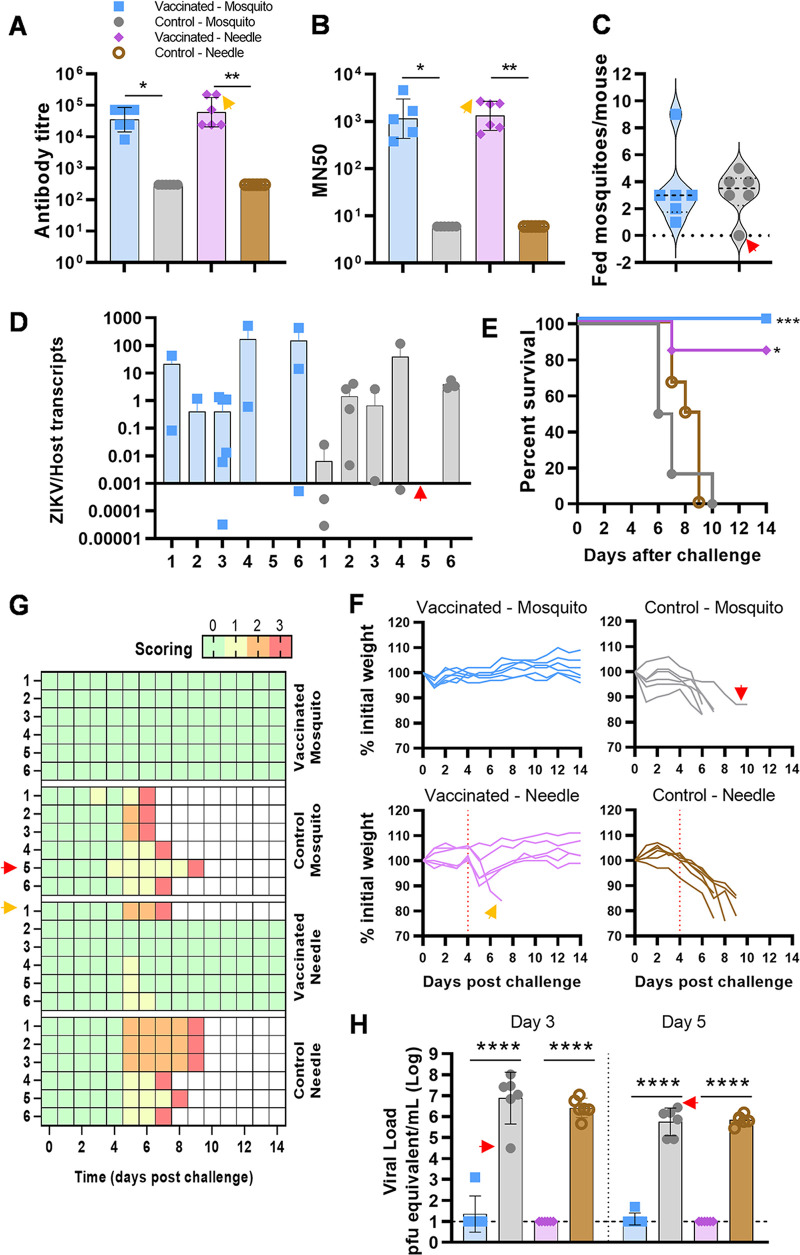
Evaluation of VLP-cvD vaccine efficacy against ZIKV MP1751 in a mouse-mosquito transmission model. (A) Anti-dimeric E antibody titers of sera collected from A129 mice immunized with VLP-cvD (blue and pink) or PBS (gray and brown). Data are geometric means with geometric SD from three independent experiments. (B) Neutralization of MP1751 ZIKV infection. The results were plotted as MN_50_ values. Data are geometric means with geometric SD from three independent experiments. (C) Number of fed mosquitoes per mouse at the end of the feeding procedure. (D) Relative quantification of ZIKV genome copies normalized to the host genome in mosquito carcasses. (E) Survival of mice in the course of the 14-day challenge. (F) Mouse scoring for signs of infection. (G) Animal body weight variations, calculated as a percentage of the initial weight. The red line indicates the day of the transmission feeding procedure (day 4). (H) Viral titers in challenged animals at days 3 and 5. Data are geometric means from all mice with geometric SD. Quantification was performed in triplicate. The dotted line indicates the limit of quantification. Red arrowheads indicate data for control-mosquito mouse 5 and orange arrowheads indicate data for vaccinated-needle mouse 1 throughout the figure. *, *P* = 0.0332; **, *P* = 0.0021; ***, *P* = 0.0002; ****, *P* < 0.0001.

We next tested the efficacy of the VLP-cvD vaccine against the African ZIKV strain MP1751, reported to induce more severe clinical signs and mortality than Asian lineages (37). For direct comparison, we also included in the study an additional group of mice for performing virus challenge via the needle route. Two groups of six A129 mice each were immunized with VLP-cvD and two groups with the PBS control, as described above. As expected, the prechallenge sera from the vaccinated mice (needle and mosquito challenged) had comparable high levels of anti-E antibodies (*P* = 1, Kruskal Wallis test) (Fig. 2A), and they efficiently neutralized MP1751 ZIKV infection of cultured cells (Fig. 2B). Animals in one vaccinated and one control group each were anesthetized and challenged by infected mosquitoes while the other two groups received a needle inoculation of 100 PFU in 100 μL via the subcutaneous route. Engorged mosquitoes collected from 5 of 6 animals of each mosquito transmission group were confirmed by RT-qPCR to carry the virus (Fig. 2C and D).

As described above, the infected animals were monitored daily for 14 days for weight loss (Fig. 2F) and scored for clinical signs (Fig. 2G). This time, consistent with the higher virulence of this African lineage ZIKV strain, each individual in the unvaccinated control groups lost weight during the course of infection and exhibited clinical signs that progressively got worse, eventually reaching a humane endpoint between 6 and 8 days on average (Fig. 2E). In contrast, all vaccinated animals survived mosquito-mediated challenge with no apparent signs of disease. Survival and protection from disease development were also excellent for needle-injected vaccinated mice, except for one individual which lost 15% of weight at day 6 and exhibited clinical signs (Fig. 2F and G). However, upon further analysis, the fatality of this animal was considered not to be related to the ZIKV infection, and this is further discussed below.

Interestingly, no engorged mosquitoes were collected from one mouse belonging to the control group infected by mosquitoes; nevertheless, this animal developed disease, reaching the endpoint 3 days after the rest of the group (from which an average of 3 engorged mosquitoes per mouse were collected) (Fig. 2, red arrowhead). This suggests that unfed/nonengorged mosquitoes can also cause a lethal ZIKV infection, most likely a result of virus inoculation during the initial probing phase of blood meal acquisition.

At days 3 and 5 postchallenge, mouse sera were collected for quantitative analysis of viremia, which showed a significant reduction of virus presence in both vaccinated groups (Fig. 2H) (*P* < 0.0001, two-way ANOVA). The comparison of the viral loads between control groups infected by mosquitoes or by needle showed no significant changes in mouse viremia when the infection was mediated by the invertebrate vector.

### Transmission blocking in immunized infected mice.

**FIG 3:**
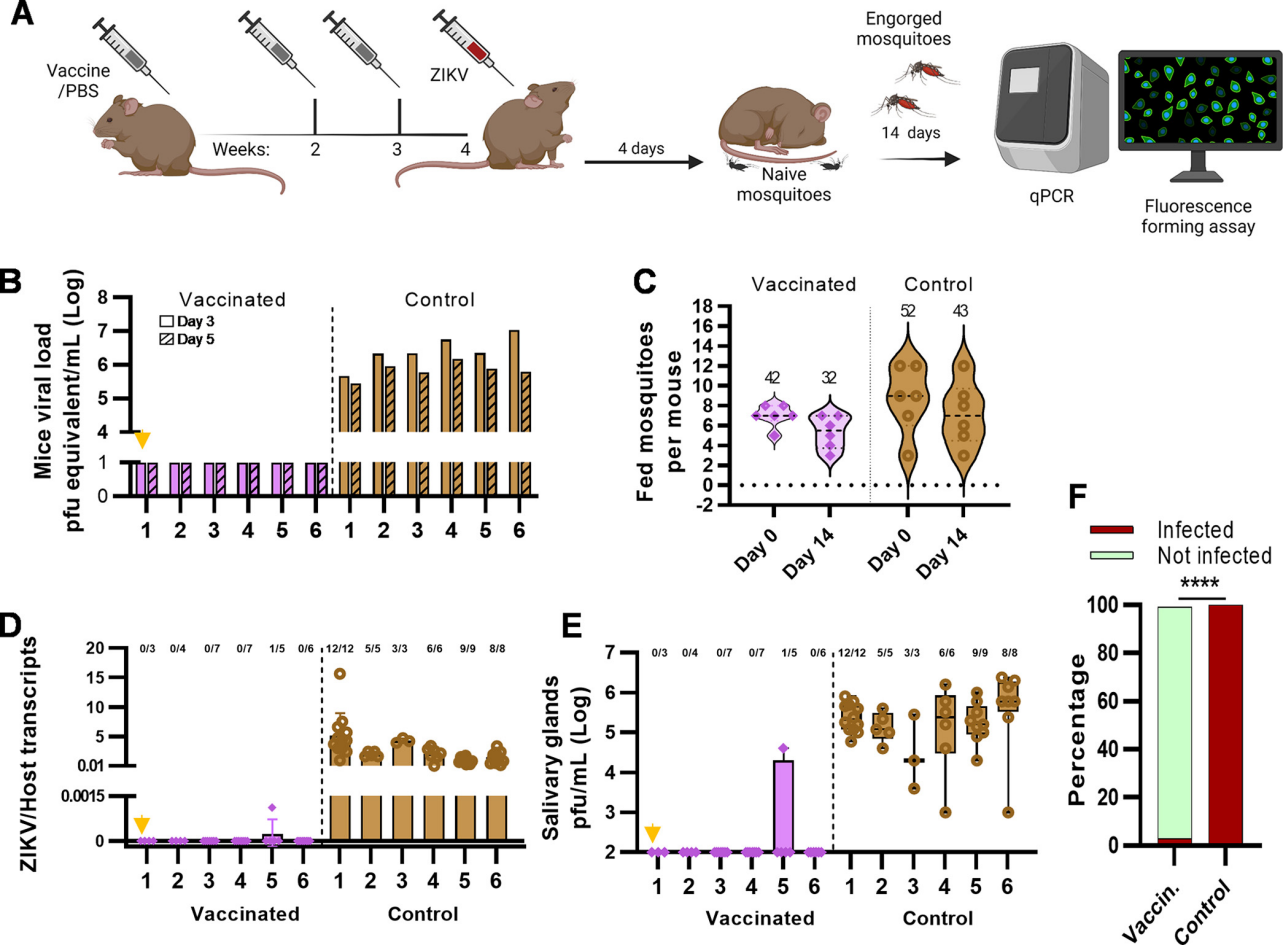
Reverse transmission from mammalian host to invertebrate vector. (A) Schematic of A129 mouse immunization, virus challenge, and subsequent postfeeding mosquito analysis. Animals correspond to the needle groups whose data are shown in Fig. 2. (B) Viral titer in challenged animals at days 3 (solid bars) and 5 (hatched bars). The *x* axis indicates individual mice. Quantification was performed in triplicate. (C) Number of mosquitoes that completed a blood meal on VLP-cvD (pink)- or PBS (brown)-injected mice. Data are the number of engorged mosquitoes at the end of the feeding procedure (day 0) and the individual surviving the 2 weeks incubation period (day 14). (D) Relative quantification of ZIKV genome copies normalized to the host genome from mosquito carcasses. (E) Quantification of viral titer in homogenized mosquito salivary glands by FFA. The data are shown as box plots with minimum and maximum values indicated; dots represent individual data points. The orange arrowhead indicates vaccinated mouse 1. (F) Percentage of infected (red) and noninfected (green) mosquitoes 14 days after feeding in vaccinated or control mice. Data are percentages of the total analyzed individuals. ****, *P* < 0.0001.

An additional objective of this study was to assess whether our vaccine was able to interfere with ZIKV transmission from an infected mammalian host to the invertebrate vector. We explored this hypothesis using the vaccinated-needle and control-needle groups of animals from the previous experiment (Fig. 2F). In compliance with the animal license regulating this study, where repetitive mosquito feedings are restricted to once a week, the needle groups were chosen over the mosquito groups, since the peak of viremia in mice was identified at day 4 postinfection using ZIKV MP1751. At the expected peak of viremia, vaccinated and control A129 mice were anesthetized, and naive mosquitoes were allowed to feed on them (Fig. 3A). Blood samples collected at days 3 and 5 after challenge confirmed the high viral load (Fig. 3B). As described above, mice experienced a reduction in body weight on the day after the anesthesia, and the vaccinated group rapidly recovered (Fig. 2F, red line) except for one individual, later culled together with the highly infected control group. However, analysis of the serum taken from this individual before and after challenge displayed an elevated titer of neutralizing antibodies (Fig. 2B, orange arrowhead) and no viremia, respectively, indicating that most probably the death was not caused by ZIKV but was a consequence of anesthesia (Fig. 3B, orange arrowhead).

Engorged mosquitoes were selected and maintained for 14 days to allow the virus to disseminate and reach the salivary glands. An average of 7 to 9 engorged mosquitoes was collected per mouse (Fig. 3C, day 0), with no significant mortality at the end of the incubation period (Fig. 3C, day 14). At day 14, mosquito salivary glands were dissected, and viral load was quantified by RT-qPCR of the carcasses (Fig. 3D) and by fluorescent focus assay (FFA) of salivary glands (Fig. 3E). Analysis showed that all mosquitoes (43/43 [100%]) (Fig. 3F) fed on infected unvaccinated control mice displayed high disseminated ZIKV load (Fig. 3D and E, brown bars). In contrast, among the mosquitoes fed on infected vaccinated mice, only one (1/32 [3.1%]) (Fig. 3F) (*P* < 0.0001, Fisher’s exact test) had acquired infection with low virus titer (Fig. 3D and E, purple). Thus, vaccination offered an excellent level of protection against viral transmission from the mammalian host to the vector, a critical aspect of the arbovirus life cycle.

## DISCUSSION

It is well established that mosquito-derived factors influence arbovirus infection in several ways, with implications for viral titer, tropism, and even disease severity. In this work, we developed a ZIKV infection model that incorporated vector-derived virus transmission to mice: this represents an epidemiologically relevant model to study real vaccine efficacy in preventing ZIKV disease and transmission. We confirmed the capacity of our vaccine candidate to protect from the disease in a mosquito-mediated challenge as well as after needle injection. In addition, by developing a mouse-mosquito transmission model, we assessed the capacity of the vaccine to inhibit viral transmission from the infected vaccinated mammalian host to the mosquito vector.

Numerous models and techniques may be used to mimic the enhancement of infection caused by a mosquito bite, including spiking the virus stock with saliva or SGE and the inoculation of virus directly into the site of mosquito bites (spot feeding). These methods are at best artificial proxies, and therefore, care needs to be taken when designing transmission blocking assays. Mosquito saliva is isolated by forcing salivation of females into a glass capillary, often containing mineral oil: the saliva obtained during this artificial collection differs qualitatively and quantitatively from the saliva that is naturally inoculated during a blood meal. SGE, on the other hand, is an extract of all the proteins present in the salivary gland tissues and not just the secreted salivary proteins/effectors that are injected into the host during mosquito probing. We considered these approaches too prone to experimental artifacts, and therefore, we opted to replicate the natural mosquito-mediated transmission cycle.

This method does not involve collection steps and has the advantage of guaranteeing that the inoculated virus has been propagated directly in the mosquito body, again mimicking the natural transmission setting. However, a caveat is the lack of a quantitative control for the inoculated dose, which may vary according to the volume of saliva inoculated by each mosquito and with the number of attempted probing events. The number of mosquitoes that completed the meal was determined by counting fully engorged females at the end of the preset feeding time. While this number varied considerably from mouse to mouse, it did not lead to a comparable variability in the infection progression, as consistent viremia and mortality were observed among the control groups. The number of fed mosquitoes per mouse was not representative of the probing events that occurred. In one instance, no blood-fed mosquitoes were collected from a control mouse (Fig. 2, control mouse 5, red arrowhead) which, nonetheless, tested positive for infection. This suggests that mere probing can lead to sufficient ZIKV transmission to induce host infection, without the uptake of a full or partial blood meal, albeit with delayed viremia and pathogenesis insurgence compared to controls.

Despite PRVABC59 being a ZIKV strain widely used in *in vivo* and *in vitro* experimental settings, in our hands it did not induce clinical signs and lethal infections in A129 mice when inoculated through a mosquito bite, although viremia was detected in blood. In a preliminary experiment, an average of 2 mosquitoes per mouse completed the meal (Fig. S1A), while an average of 4 to 6 engorged mosquitoes per mouse were collected for testing vaccine efficacy (Fig. 1D). Keeping in mind that the number of engorged mosquitoes is only a fraction of the probing mosquitoes and that lethal infection was observed in the absence of complete meals, this suggests that the lack of pathogenicity in this case was not dependent on an inadequate mosquito feeding attempt. In addition, PRVABC59 has been previously associated with loss of pathogenicity in single-knockout animals due to mutations acquired after a low number of passages in cell culture (38, 39).

Consistent with these studies, we confirmed ZIKV PRVABC59 lethality in AG129 mice in a challenge evaluation that compared side-by-side mosquito- and needle-mediated inoculation. In this specific instance, the higher susceptibility of the AG129 mouse strain is likely caused by the double-knockout genotype; however, differences in host susceptibility also point to the importance of the interactions between the host genetic background and the viral strain. Despite being associated with the most concerning human outbreaks, paradoxically, ZIKV strains belonging to the Asian lineage show reduced *in vitro* replication and *in vivo* pathogenicity compared to the African lineages (10, 40). Also, ZIKV strains belonging to the African lineage are reported to be more efficient in transmission than strains from Asian lineage (41). Taking into consideration all these variabilities, we tested the efficacy of our vaccine candidate using the most appropriate available vector-host transmission system, combining a commonly used mouse strain, a highly pathogenic ZIKV strain (such as the African strain MP1751), and a highly susceptible lab-adapted A. aegypti mosquito strain having the globally invasive genotypic background.

While ZIKV infection can be sustained by the sylvatic cycle, with nonhuman primates as a mammalian reservoir for the virus, densely human-populated environments, combined with the remarkably anthropophilic behavior of A. aegypti, strongly support ZIKV transmission in the urban cycle. In this study, by allowing naive mosquitoes to feed on viremic mice, we also assessed the vaccine’s capacity to interfere with the transmission cycle, inhibiting the passage of the virus from vaccinated individuals to naive mosquito vectors. A complete viral transmission with high titers was observed in mosquitoes fed on control nonvaccinated mice, while only one individual fed on a vaccinated mouse showed a mild ZIKV infection in the carcass. When the potential of mosquitoes to transmit the virus was observed by analyzing the titer of live viral particles in the SG, the vaccine was also able to efficiently interfere with mosquito capacity to spread the virus.

In conclusion, our findings confirm the capacity of the vaccine candidate VLP-cvD to protect mice against ZIKV infection when the infection occurs through the bite of a mosquito vector. The vaccine is also capable of interfering with ZIKV spread by blocking the transmission of the virus from an infected mammalian host to uninfected mosquitoes. This study underlines the importance of considering the complexity of the vector-host-virus interaction when developing and evaluating disease control interventions and further supports the potential of VLP-cvD as a vaccine candidate, making it worthy of further development for clinical use.

## MATERIALS AND METHODS

### Animal ethics.

All animal research described in this study was approved by the University of Glasgow Animal Welfare and Ethical Review Board and was carried out under United Kingdom Home Office Licenses, P9722FD8E, in accordance with the approved guidelines and under the UK Home Office Animals (Scientific Procedures) Act 1986 (ASPA).

### Cell lines and virus strain.

Expi293F embryonic human kidney cells were maintained and transfected in Expi293 expression medium (Thermo Fisher Scientific) as per the manufacturer’s protocol. Vero-furin cells (kindly supplied by Theodore C. Pierson) (42) were grown in Dulbecco’s modified Eagle’s medium (DMEM) (Life Technologies) containing 7% fetal bovine serum (FBS) (Life Technologies), 10 μg/mL of blasticidin (InvivoGen), and penicillin-streptomycin (Gibco). The virus was serially passaged in *A. albopictus* C6/36 cells: the infected supernatant was harvested, concentrated using Amicon Ultra-15 filters (Millipore, IRL), and titrated via fluorescent focus assay (FFA), as described below. ZIKV PRVABC59 (kindly supplied by BEI Resources; accession number KX087101) and ZIKV MP1751 (005V-02871; kindly supplied by Public Health England; accession number KY288905.1) were used for microneutralization, mosquito injections, and animal challenges.

### VLP-cvD production and purification.

VLP-cvD were produced in Expi293F cells at 28°C following transfection with a plasmid expressing ZIKV prM-E-A264C and purified as described by De Lorenzo et al. (11) by performing, in sequence, 20% sucrose cushion centrifugation, discontinuous 10 to 30% sodium potassium tartrate density gradient separation, and size exclusion chromatography with elution in PBS buffer. Eluted fractions were concentrated by ultrafiltration through Amicon Ultra-15 filters (100 kDa; Merck Millipore).

### Mouse immunization and ZIKV challenge.

A129 mice or AG129KO mice (129 Sv/Ev background; Marshall BioResources) at 4 weeks of age were immunized subcutaneously with either VLP-cvD or PBS, both adjuvanted with 1% AddaVax (InvivoGen). Needle challenge was performed by subcutaneous injection of PRVABC59 ZIKV (10^4^ PFU) or MP1751 ZIKV (100 PFU) in 100 μL 2% FBS-DMEM. Mosquito challenge was performed as described below. Pre- and postchallenge blood samples were collected for antibody titration, microneutralization assay, and determination of serum viral load. After challenge, animal weight change and signs of infection were monitored daily and scored for 14 days. The scoring system was as follows: 0, no signs of distress or disease; 1, one sign of distress; 2, two signs of distress or mild disease; and 3, more than two signs of severe disease or loss of 15% of body weight. A score of 3 was considered the humane endpoint, and mice were culled. Any individual mouse reaching a clinical score of 3 or losing more than the 15% of its initial weight was euthanized. Surviving mice were euthanized at 14 days postchallenge.

### ELISA for antibody titration.

Recombinant biotinylated sE-cvD protein was expressed at 28°C using an ExpiFectamine 293 transfection kit (Thermo Fisher Scientific). Cell supernatant was harvested and dialyzed. Biotinylated proteins were captured in enzyme-linked immunosorbent assay (ELISA) plates precoated with 5 μg/mL of avidin (Sigma) in Na_2_CO_3_-NaHCO_3_ buffer (pH 9.6) and subsequently blocked with PBS containing 0.05% Tween 20 (PBST) and 1% bovine serum albumin (BSA; Sigma). Serial dilutions of mouse sera were tested for binding to the biotinylated proteins, and the bound antibodies were detected using horseradish peroxidase (HRP)-conjugated anti-mouse IgG A4416 (Sigma) and 3,3′,5,5′-tetramethylbenzidine (TMB) substrate (Life Technologies).

### Microneutralization assay.

The microneutralization assay was performed as described by Lopez-Camacho et al. (15). Briefly, Vero-furin cells were seeded the day before the experiment at a density of 7 × 10^3^/well in 96-well plates. Threefold serially diluted mouse sera were first incubated at 37°C for 1 h with 100 PFU/well ZIKV. The serum-virus mix was then used to infect cells. After 1 h of incubation at 37°C, 100 μL of medium was added to each well. At day 3 postinfection, cells were lysed in lysis buffer (20 mM Tris-HCl [pH 7.4], 20 mM iodoacetamide, 150 mM NaCl, 1 mM EDTA, 0.5% Triton X-100, and cOmplete protease inhibitors), and the viral E protein was quantitated by sandwich ELISA (see below). The amount of E protein detected correlates with the level of virus infectivity, which is presented as percent ZIKV infectivity relative to the control (i.e., virus not preincubated with immune sera). Virus infectivity values were plotted using GraphPad Prism 6, and nonlinear regression (curve fit) was performed for the data points using log(inhibitor) versus response (variable slope) to determine MN_50_ titers. The MN_50_ titer was defined as the serum dilution that neutralized ZIKV infection by 50%.

### Sandwich ELISA for quantification of ZIKV infectivity.

ELISA plates were coated with 3 μg/mL of purified pan-flavivirus monoclonal antibody (MAb) D1-4G2-4-15 (ATCC HB112TM) in PBS, incubated overnight at room temperature, and blocked for 2 h at room temperature with PBST and 2% skim milk powder. After washing with PBST, ZIKV-infected cell lysates were added and incubated for 1 h at room temperature. Wells were washed with PBST, incubated with anti-ZIKV E rabbit polyclonal R34 IgG (9) at 6 μg/mL in PBST for 1 h at RT, and then washed again. Antibodies bound to ZIKV envelope protein were detected using HRP-conjugated anti-rabbit IgG 7090 (Abcam) and TMB substrate (Life Technologies). The MN_50_ titer was defined as the serum dilution that neutralized >50% of ZIKV and was determine using GraphPad Prism 9, and a nonlinear regression (curve fit) was performed for the data points using log (inhibitor) versus response (variable slope).

### Mosquito rearing.

The A. aegypti wild-type line used was colonized from Selangor State, Malaysia, in the 1960s. Colonies were maintained under standard rearing conditions, at 27°C and 70% relative humidity with a 12-h light/dark cycle. Larvae were fed on tropical fish pellets (Tetramin; Tetra, Melle, Germany), and adults were maintained with 5% sucrose solution *ad libitum*. Blood meals to maintain the colony were provided using an artificial blood-feeding system (Hemotek, UK) using human blood (Scottish National Blood Transfusion Service, UK). Eggs were collected on a wet filter paper (grade 1 filter paper; Whatman plc, GE Healthcare, UK), desiccated for 5 days, and later hatched in deionized water containing 1 g/L bovine liver powder (MP Biomedicals, Santa Ana, CA, USA).

### Mosquito infection.

Five-day-old female mosquitoes were infected by intrathoracic injection of 69 nL of a 10^6^-focus-forming-unit (FFU)/mL dilution of ZIKV using a Nanoject II (Drummond Scientific, USA) hand-held microinjector. Injected mosquitoes were immediately transferred into a climatic chamber at 27°C with 70% relative humidity and a 12-h light/dark cycle for recovery. ZIKV-injected females were maintained on sugar solution for 7 days prior to mouse infection.

### Mosquito feeding on mice.

Mice were transferred from the animal house to the insectary, where they were anesthetized using a ketamine-medetomidine cocktail (Domitor) based on their body weight (ketamine, 50 mg/kg; medetomidine, 0.5 mg/kg). Once anesthesia was effective, each mouse was transferred for 20 min onto the organdy cover of a mosquito-containing cardboard cup, with the eyes protected while the mosquitoes fed through the net. At the end of the feeding, the anesthetic effects were reversed with atipamezole (Antisedant; 1 mg/kg), and the condition of the mice was monitored for the rest of the day. After feeding, the number of fully engorged mosquitoes was recorded for every mouse. Cardboard cups with fed mosquitoes were placed back in the climatic chambers.

### RT-qPCR for mouse viremia.

Viral RNA was extracted from postchallenge sera with a QIAamp viral RNA minikit (Qiagen). Viral load was measured by RT-qPCR using a one-step SYBR Primescript RT-PCR kit II (TaKaRa). Cycle threshold (*C_T_*) values from serum samples were used to calculate serum viral load according to regression equation built by a set of standard viral RNA extracted from dilutions of known titer virus preparation. The primer pair for the PRVABC59 ZIKV gene was 5′-TTGGTCATGATACTGCTGATTGC-3′ (forward) and 5′-CCTTCCACAAAGTCCCTATTGC-3′ (reverse), while for the MP1751 ZIKV gene, we used 5′-ACTTCCGGTGCGTTACATGA-3′ (forward) and 5′-GGGCTTCATCCATGATGTAG-3′ (reverse).

### RT-qPCR for mosquitoes.

RNA was extracted using TRI reagent (Sigma-Aldrich, MO, USA). cDNA was synthesized using 1 μg of total RNA and all-in-one cDNA synthesis SuperMix (Biotool, Houston, TX, USA). qRT-PCRs were performed on a 1:20 dilution of the cDNAs. Virus levels were normalized to the transcripts of the mosquito RpS17 housekeeping gene (RpS17-F, 5′-CACTCCCAGGTCCGTGGTAT-3′; RpS17-R, 5′-GGACACTTCCGGCACGTAGT-3′).

### Viral quantification in mosquito salivary glands.

Mosquito salivary glands were dissected and sampled in DMEM supplemented with 2% FBS. After homogenization, 10-fold serial dilutions of each solution were transferred onto a monolayer of Vero-furin cells for viral quantification by FFA. The primary antibody for Zika was mouse monoclonal antibody DIII1B (11); the secondary antibody was Alexa Fluor 488-conjugated goat anti-mouse immunoglobulin (A-11001; Thermo Scientific, Waltham, MA, USA). A Celigo imaging cytometer (Nexcelom Bioscience, Lawrence, MA) was used for imaging plates. Fluorescent foci were quantified by eye (from dilutions with less than 100 foci), and virus titers calculated and expressed in FFU per milliliter.

### Statistical analysis.

All graphs and statistical analyses were produced using GraphPad Prism 9 (GraphPad Software Inc., San Diego, CA, USA). A Shapiro-Wilk test was used for assessing normality distribution of data, and parametric and nonparametric tests were selected accordingly for antibody and virus titers. Multiple comparisons were calculated using the Bonferroni method for *P* value adjustment. Statistical analysis of survival was performed using a log-rank (Mantel-Cox) test with a 95% confidence interval. Statistical analysis of viremia was performed using a 2-sided ANOVA, with Tukey’s pairwise comparison at a 95% confidence interval.
