# Supplementary figures and images for: Evaluation of an Engineered Zika Virus-Like Particle Vaccine Candidate in a Mosquito-Mouse Transmission Model
Source: mSphere. 2023 Feb 22;8(2):e00564-22. doi: 10.1128/msphere.00564-22 (PMC10117074; doi:10.1128/msphere.00564-22)

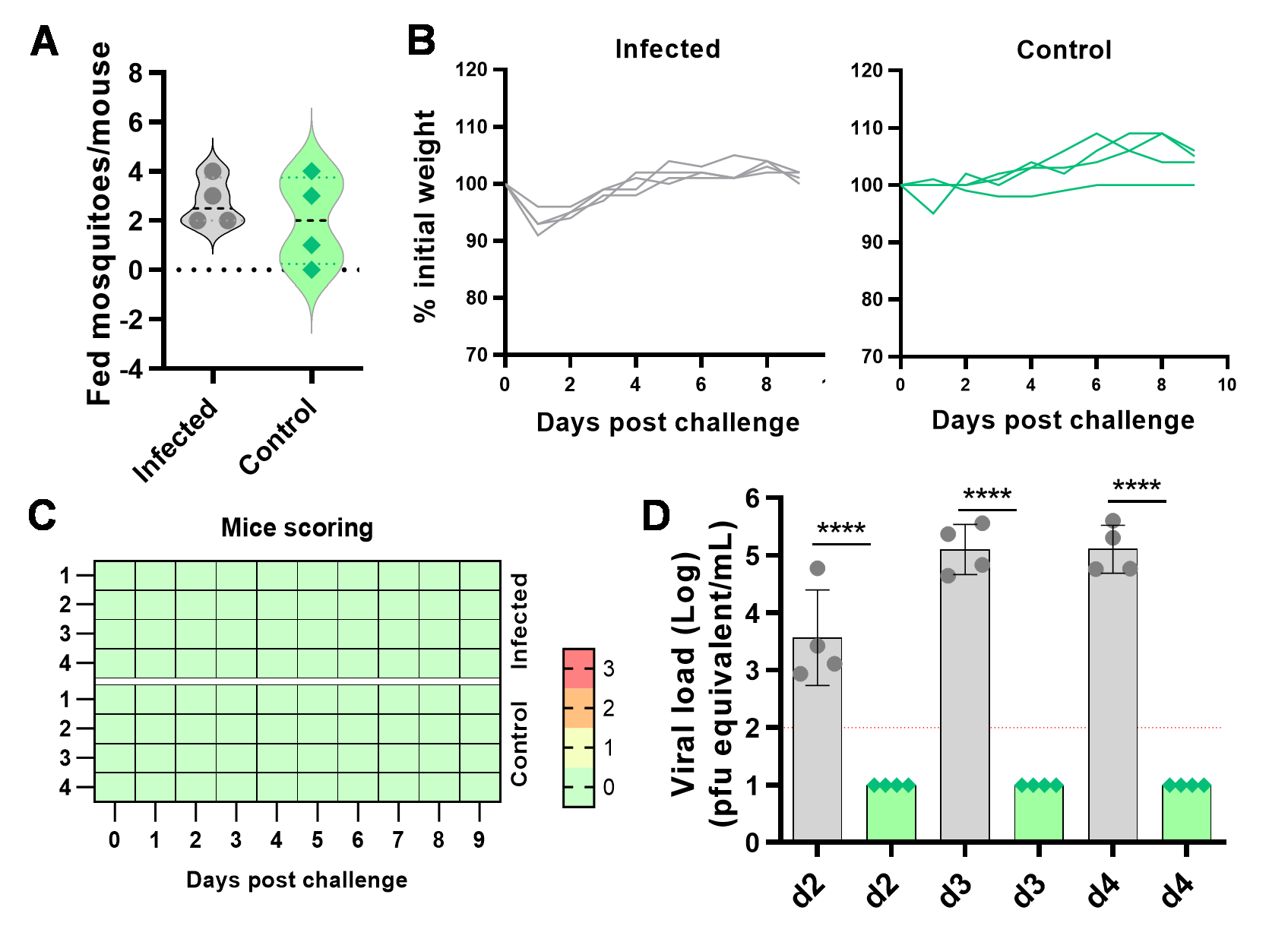

Supplement: FIG S1 [file msphere.00564-22-s0001.tif]

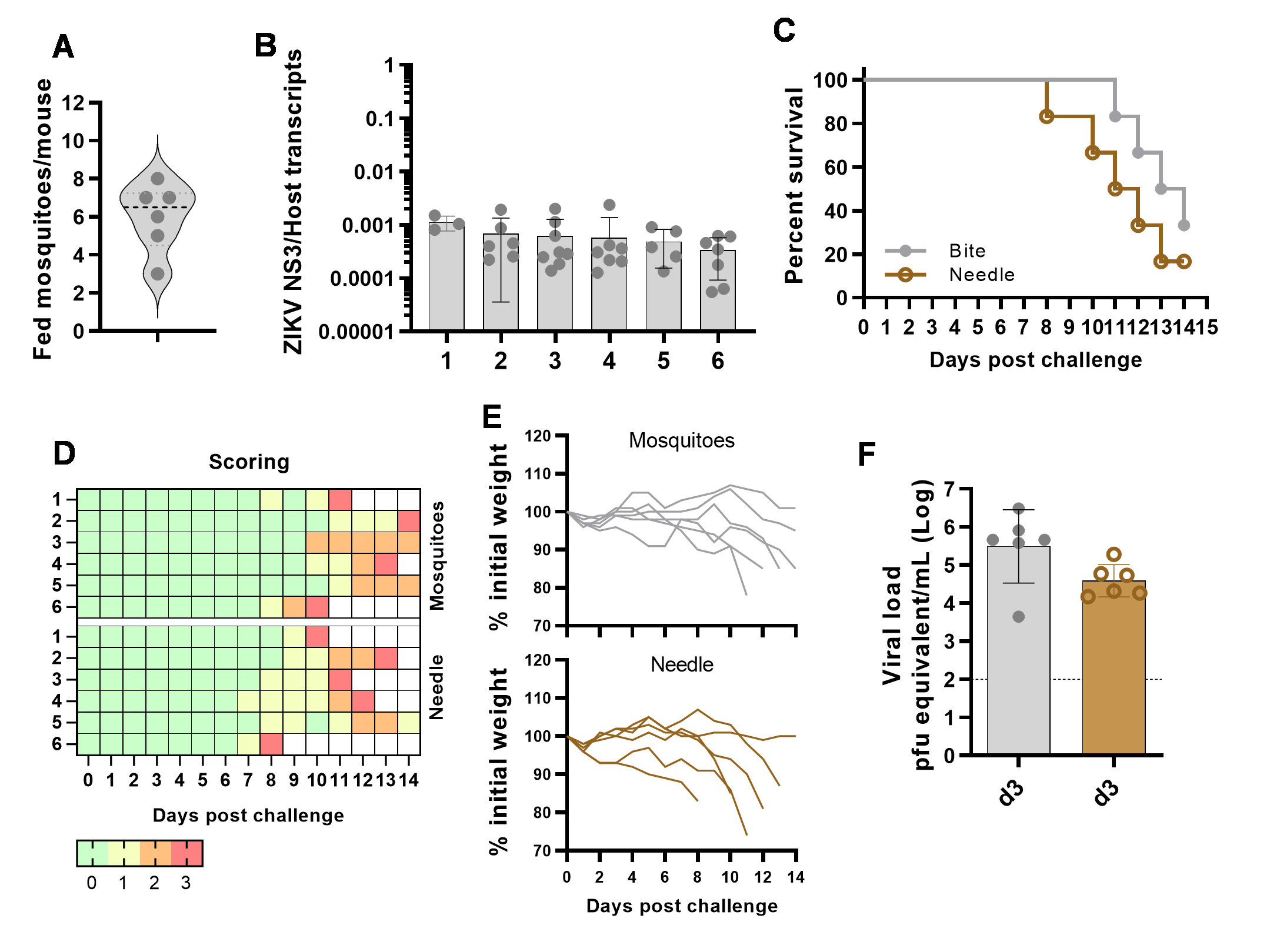

Supplement: FIG S2 [file msphere.00564-22-s0002.tif]
